# Relationship between Emotional Intelligence, Social Skills and Peer Harassment. A Study with High School Students

**DOI:** 10.3390/ijerph17124208

**Published:** 2020-06-12

**Authors:** Rubén Trigueros, Elena Sanchez-Sanchez, Isabel Mercader, José M. Aguilar-Parra, Remedios López-Liria, María José Morales-Gázquez, Juan M. Fernández-Campoy, Patricia Rocamora

**Affiliations:** 1 Department of Language and Education, University of Antonio de Nebrija, 28015 Madrid, Spain; rtr088@ual.es; 2 Department of Psychology, University of Almeria, 04120 Almeria, Spain; helenaasanchezsanchez@gmail.com; 3 Department of Psychology, Hum-878 Research Team, Health Research Centre, University of Almeria, 04120 Almeria, Spain; 4 Health Research Centre, Department of Nursing, Physiotherapy and Medicine, University of Almeria, 04120 Almeria, Spain; rll040@ual.es (R.L.-L.); rocamora@ual.es (P.R.); 5 Department of Nursing, University of Las Palmas de Gran Canaria (ULPGC), Juan de Quesada, 30, 35001 Las Palmas de Gran Canaria, Spain; mariajose.morales@ulpgc.es; 6 Department of Education, University of Almeria, 04120 Almeria, Spain; jfc105@ual.es

**Keywords:** emotional intelligence, social skills, bullying, adolescence, high school

## Abstract

The objective of this study was to analyse the relationship between emotional intelligence and social skills, and how these two variables influence bullying. In this study, 912 Spanish high school students, 471 boys and 441 girls aged 14–16 years, participated, who were administered the Spanish version of the Trait Meta Mood Scale 24, the “*Bateria de socialización BAS-3*” and the Peer Harassment Questionnaire. To analyse the results, a structural equation model was made. The results reflected a positive relationship between emotional intelligence and social skills (B = 0.44, *p* < 0.001), and a negative relationship with respect to bullying (B = −0.56, *p* < 0.001). In turn, social skills reflected a negative relationship with respect to bullying (B = −0.38, *p* < 0.001). These results reflect the need to implement educational programs focused on the development of emotional intelligence in the classroom, as a means to try to stop bullying behaviours in the classroom.

## 1. Introduction

In adolescence, the process of socialization becomes especially important. It is a transitional stage, during which young people not only experience physical, cognitive and emotional changes, but also changes in social expectations and behavioural patterns [1]. However, several studies have found the beginning of the incidence of bullying during this period, especially during the high school stage [2]. This hostile environment may generate, in bullies, an entrenchment in the future of behaviours linked to harassment and abuse during the adult stage; they may also develop social insensitivity and indifference to the suffering of others [3]. On the other hand, students who self-identify as victims self-evaluate the effects of bullying from a negative perspective, negatively affecting their social, emotional, academic, somatic and family self-concept. In addition, the strategies that the person uses to cope with the harassment are related to emotions, which could determine the chronification, or not, of the victimization [4,5]. Thus, emotional intelligence (EI) can play a very important role in the social and behavioural performance of adolescents. Results from various studies highlight that people with higher levels of EI are those who show greater empathy, which will facilitate their involvement in positive social relationships with their peers [6,7,8]. Therefore, this study aims to analyse the relationship between emotional intelligence and social skills, and the influence that these two variables have on peer harassment in high school students.

### 1.1. Emotional Intelligence

EI is a construct that has gained enormous interest in recent decades. One of its main areas of study has been the influence it has on interpersonal relationships by contributing to optimal social functioning [9]. There are two major models of IE: the mixed model [10] and Bar-On [11], and the ability model, represented mainly by Mayer, Salovey and Caruso [12]. The mixed model considers EI as a set of characteristics that are linked to various cognitive skills, stable personality, motivational aspects and social-emotional competencies. On the other hand, the skills model focuses on how the individual emotionally processes information and the skills related to this processing. From this model, it is assumed that emotion and intelligence are related. Thus, this perspective understands EI as: (a) a capacity to value, understand, perceive and express emotions accurately; (b) a capacity that facilitates thinking through the access and generation of feelings; (c) a capacity that promotes emotional growth through the regulation of emotions [13]. This model makes it possible to study the role that emotional skills play in social functioning.

Recent studies have shown a positive relationship between emotional intelligence and social skills. The studies carried out from the perspective of Mayer and Salovey’s [13] model assume that a student with a high score in EI has been attributed to be a person more skilled in the perception and understanding of other people’s emotions and possesses better regulation skills, which will support the development of more appropriate behaviour patterns with his or her peers [13]. In addition, different studies have shown that students with a high IEs have fewer physical symptoms [14], less depression and anxiety, greater interpersonal satisfaction, better self-esteem, and greater use of active coping strategies to solve their problems [15]. In addition, students who are stressed by academic issues perceive this stress as non-threatening and have low cortisol and blood pressure levels [16], even being able to return to emotional homeostasis faster [17].

On the other hand, it has been found that deficiencies in EI skills affect people in any context, especially students in and out of school [17,18]. Liau et al. [19] found that high school students with low levels of emotional intelligence show greater predictability toward aggressive and/or criminal behaviour; and Zimmerman [20] concluded that bullies show a very low emotional intelligence, especially in those aspects related to social skills, self-control and empathy.

Therefore, studies have shown that students with a high level of EI will possess more adequate behaviour patterns with their peers, while students with a low level of EI will show higher scores in the occurrence of disruptive behaviours. However, at present, there is little evidence of studies that have analysed the relationship between emotional intelligence and bullying behaviour suffered by the student, as well as social skills. Thus, the present study aims to answer the relationship between these three variables.

### 1.2. Social Skills

Social skills are defined as capabilities or aptitudes employed by an individual when interacting with other people on an interpersonal level [21]. In this sense, they play an important role, not only in socialization, but also for individualization, since they allow knowledge of oneself and others, which contributes to the formation of self-concept [22]. They also promote the development of some aspects of social knowledge and certain behaviours, strategies and skills, such as empathy, reciprocity and role-taking, which are important for interacting with others [23]. In addition, feedback from others supports self-control and the self-regulation of one’s own behaviour, since peers act as control agents by punishing or reinforcing certain behaviours [24]. Social skills are also a source of enjoyment and provide emotional support, since peer relationships are sources of intimacy, help, support, affection, a sense of inclusion, and feelings of belonging and acceptance. In turn, they facilitate the learning of sexual role and values, as well as moral development. Taking all this into account, we can conclude that social skills have a positive function in the relationship between equals.

Depending on the quality of the socialization process in the family, school and in peer groups, the subject will acquire more or less positive social skills. These social skills depend on a process of learning experiences, which do not always lead to socially appropriate behaviour. According to Trinidad and Johnson [25], adolescents who are emotionally intelligent are better able to detect emotional pressures that may arise in class or from their peers. They are able to cope with the differences between their own emotions and those of their peers, unlike adolescents, who have less control over their emotions. Therefore, the former will be able to have enough self-control not to fall into self-destructive behaviour. In relation to the disruptive behaviours of secondary school students, different studies have shown that the appearance of disruptive behaviours is related to an emotional deficit [26]. That is, it is more likely that an adolescent with a low emotional intelligence deficit will show antisocial and aggressive behaviours [27]. These behaviours are not always towards other people, but they also focus it towards themselves, that is, self-destructive behaviours, such as tobacco and alcohol consumption, and behaviours that are related to low IQ [28,29]. However, current studies have focused on the reasons for bullying behaviours (e.g., family problems, delinquency, lack of self-control, etc.), so it is important to analyse the social skills that may be an indication of bullying behaviours, or at least those that can be related to bullying.

### 1.3. Peer-to-Peer Harassment

In recent decades, bullying has become a major social phenomenon. A study carried out with Spanish students indicates a level of participation in school bullying of 16.2%, with 8.1% aggressors, 6.8% victims and 1.3% both victims and aggressors [30]. 

Bullying is characterized by a series of repeated aggressions (verbal or physical) by one student or group towards another, generating a dynamic of domination-submission between the aggressor and his or her victim [31]. Students who participate in this behaviour present an inadequate psychological adjustment. Several factors and cognitive-emotional processes accompany this phenomenon. We have studied the role of the different elements of emotional processing-perception, understanding and/or regulation—as possible predictors of victimization and peer rejection.

Studies that have analysed the emotional consequences of harassment on the victim have found a wide spectrum of negative emotions that vary from one victim to another [32,33]. Peer violence has harmful consequences for all those involved, affecting health, quality of life, well-being and proper development of the person [34], and can cause stress and anxiety for the victim.

According to Serrano [35], there is no specific reason why some children or young people become aggressors or victims; rather, there is a set of family, school, social, cultural and personal variables that can prevent or develop bullying behaviour. Those with more aggressive behaviour are clearly more likely to become bullies, and those who are more shy are more likely to become victims. In addition, some studies suggest that a good deal of bullying problems may be caused by poor emotional management [36]. In this sense, it is considered that EI could become a protective variable against bullying. Furthermore, different authors consider that EI is related to the social component, and more to social adjustment and the control of emotions and feelings. 

### 1.4. Objectives and Hypotheses

Taking into account the above, this study aimed to analyse the relationship of emotional intelligence in high school students with social skills, and how these two variables influence bullying among peers. The hypothesis are: (a) emotional intelligence positively predicts social skills; (b) emotional intelligence and social skills negatively predict bullying among peers.

## 2. Materials and Methods 

### 2.1. Participants

The sample for this study was: 471 boys and 441 girls (*N* = 912), aged between 14 and 16 years (Mean = 15.01; Standard Deviation = 0.75), from several educational centres in a Spanish province. The classes respected the equality of rights and duties among the students. The sampling method has been non-probabilistically incidental, depending on those centres to which access was obtained.

### 2.2. Measurements

#### 2.2.1. Emotional Intelligence

The Trait Meta Mood Scale (TMMS-24; [37]) was used, specifically the Spanish version of Fernandez-Berrocal, Baena-Extremera and Ramos [38]. This questionnaire is composed of 24 items that are distributed among 3 factors: attention, clarity and repair. The instrument is scored on a Likert scale from 1 (no agreement) to 5 (full agreement). 

In this study, Cronbach’s alpha values of 0.81, 0.87 and 0.83 were obtained, respectively.

#### 2.2.2. Peer-to-Peer Harassment

The Magaz, Chorot, Santed, Valiente and Sandin [39] Peer Harassment Questionnaire was used. It consists of 39 items common to boys and girls, which refer to different bullying behaviours that children may suffer from other boys/girls. From this scale, the following forms of peer bullying (subscales) can be evaluated separately: (A) verbal abuse (11 items), (B) direct social exclusion (5 items), (C) threats (4 items), (D) cyberbullying (4 items), (E) indirect social exclusion (4 items), (F) object-based aggression (3 items), and (G) physical abuse (8 items). There is a different specific part for boys and girls, which consists of 10 items, 5 for boys and 5 for girls. The content of the specific items for boys refers to being verbally abused due to lack of physical attractiveness or interest in girlish activities or things. In the case of girls, the content of the items refers to being verbally abused due to lack of physical attractiveness or having a high interest in stereotypical activities of the other sex. Students had to indicate their response by means of a Likert scale from 1 (never) to 3 (many times). 

In this study, the factors obtained Cronbach’s alpha values of: 0.84 for verbal abuse, 0.87 for direct social exclusion, 0.80 for threats, 0.79 for cyberbullying, 0.77 for indirect social exclusion, 0.83 for object-based aggression, and 0.82 for physical abuse.

#### 2.2.3. Social Skills

The version of Silva and Martorell [40] was used. This questionnaire is made up of 75 items, which are distributed among six factors: consideration with others, self-control in social relations, social withdrawal, social anxiety/shyness, sincerity, and leadership. Students must answer by choosing their response, to different situations of daily life, between two options (YES–NO). 

In this study, the factors obtained a Cronbach’s alpha values of: consideration with others of 0.76, self-control in social relations of 0.78, social withdrawal of 0.82, social anxiety/shyness of 0.80, sincerity of 0.86 and leadership of 0.78.

#### 2.2.4. Procedure

Before the study began, an informed consent form was prepared and given to the school and the parents of the students who wanted to participate in the study, due to the minority age of the latter. Subsequently, before giving them the informed consent, the school representatives and the parents were informed of the objective of the study and the duration of the collection of information through the questionnaires. The students, who wished to participate in the study, were also informed of the objectives of the research. 

The data collection was done before the beginning of the classes, respecting the ethical standards established by the American Psychological Association’s (Ref. UALBIO 2019/014) and Helsinki Protocol.

### 2.3. Data Analysis

For this study, several analyses were carried out. Firstly, the mean and standard deviation of each of the factors in the study were analysed, as well as the Pearson correlations. Secondly, Cronbach’s alpha was calculated to analyse the reliability of the factors; for these analyses the statistical software SPSS version 25 (IBM, Armonk, NY, USA) was used.

A structural equation model (SEM), using AMOS version 20 statistical software (IBM, Armonk, NY, USA), was used to analyse the predictive relationships between the variables of the study.

**Figure 1:**
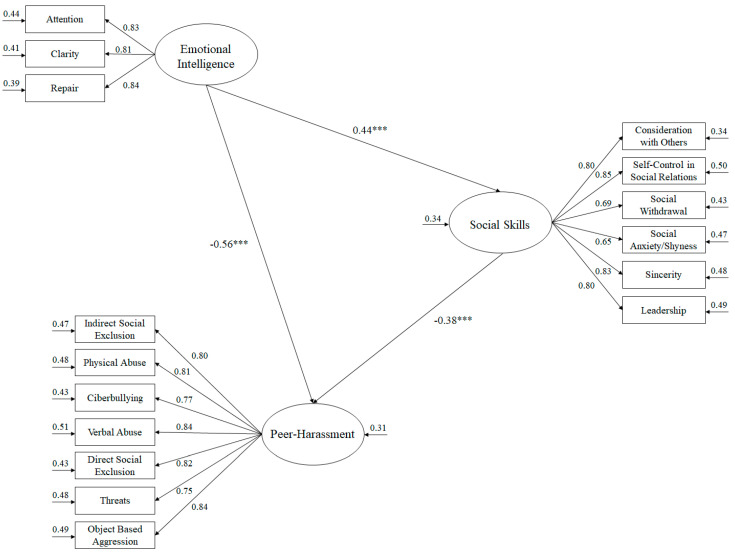
Structural equation model. The explained variances are shown on the small arrows. All parameters are standardized and statistically significant. Note: *** *p* < 0.001.

A structural equation model was used to analyse the predictive relationships of the hypothesized model (see Figure 1). For this purpose, the bootstrapping procedure was used together with the maximum likelihood method. The estimators were considered robust, despite the lack of normality [41]. 

Thus, the adjustment indexes taken into account to reject or accept the model tested were the following [42,43,44,45,46] (see Table 1).

## 3. Results

### 3.1. Preliminary Analysis

Table 2 shows the descriptive statistics with means, standard deviations and bivariate correlations. The correlations were shown to be positive between emotional intelligence and social skills. In contrast, the correlations were negative between peer harassment and social skills and emotional intelligence.

### 3.2. Structural Equation Modelling

To test the predictive model, the number of latent variables was reduced. This step is performed when the study sample is not too large compared to the number of variables that make up the model [47]. Specifically, the latent variables used were: emotional intelligence, which included three indicators; attention, clarity and repair, as suggested [37]; social skills, which included consideration for others, self-control in social relationships, social withdrawal, social anxiety/shyness, sincerity and leadership [39] and finally, bullying behaviours among peers, which included verbal abuse, direct social exclusion, threats, cyberbullying, indirect social exclusion, object-based aggression and physical abuse [40]. 

The fit indexes showed adequate psychometric properties for the hypothesized model (Figure 1) [42]: χ2 (101, *N* = 912) = 301.54, χ2/df = 2.98, *p* < 0.001, TLI = 0.96, IFI = 0.96, CFI = 0.96, RMSEA = 0.062. (IC 90% = 0.057–0.069), SRMR = 0.058. Predictive relationships between study variables were evaluated using standardized regression weights.

## 4. Discussion

The aim of this study was to analyse the relationship between emotional intelligence and social skills in Spanish high school students, and how these two variables influence bullying among peers. In this sense, if students are able to recognize their own and other people’s emotions, and are better able to regulate their emotions, they will have more tools to generate adequate behaviour patterns with their peers [12].

The results of this study show a positive association between emotional intelligence and social skills, and a negative association between these two variables and bullying behaviour among peers. These results are comparable to similar studies in the context of teaching [48,49]. In this regard, a study by Mayer, Caruso and Salovey [12], in which they administered the “Multifactor Emotional Intelligence Scale” (MEIS; [50]), to a sample of 183 American university students, found that the most emotionally intelligent people were those who desired and sought greater social involvement, both in general and in intimate social relationships. These results are in line with other research (e.g., Tiwari and Bhat, [8]; Mayer, Salovey and Caruso, [37]), which highlights that people with higher levels of EI are, in turn, those who show greater empathy, which will facilitate their involvement in positive social relationships with their peers. Thus, Gorostiaga, Balluerka and Soroa [51] found positive and significant correlations between empathy and the three dimensions of EI, namely perception, understanding and regulation, evaluated by the TMMS-23. Similarly, Salguero, Fernandez-Berrocal, Ruiz-Aranda, Castillo and Palomera [52], showed that adolescents with a greater ability to recognize the emotional states of others reported better social relationships with their peers. Therefore, the results of the present study are similar to the results shown in previous studies, as emotional intelligence can contribute to the development of social skills in general and to better relationships with their peers. Therefore, both factors can be considered as predictors of optimal social functioning in adolescents [53].

The results have shown that emotional intelligence is a negative predictor of bullying among peers in adolescents. These results are similar to some studies suggesting that a good part of bullying problems could have their origin in a bad emotional management [30]. It is considered that emotional intelligence could become a protective variable against bullying in the classroom, since it allows good management and emotional expression, and thus, could reduce cases of bullying. In addition, another study suggests that the phenomenon of bullying is related to one’s emotions. In this sense, Garaigordobil and Oñederra [54] found that adolescents who had suffered bullying behaviours, had low levels of emotional intelligence, the same way it happened with the aggressors. Therefore, an optimal development of emotional intelligence will provide a control over emotions that will prevent violent behaviour by the bullies. That is why the existence of a relationship between emotional intelligence and bullying among equals is considered one.

In addition, the results of this study have shown that social skills are a negative predictor of peer-to-peer bullying in adolescents. Current descriptions of social skills summarize them in a set of habits our behaviours, also in our thoughts and emotions, which provide us with appropriate interpersonal relationships, feelings of well-being in relationships with others, the achievement of our goals without anyone hindering us and effective communication with everyone around us [55]. Therefore, social skills are essential, not only because of their relational dimension, but also because of their influence on other areas of the adolescent’s life. According to positive psychology, social skills operate as a protective factor and constitute a salugenic resource in early adolescence [56].

The present study shows the importance of promoting emotional education, since it is one of the most useful forms of prevention against coexistence problems. In this sense, all actions should always be aimed at creating a positive climate; for this reason, emotional education becomes an essential aspect for facing problems in relationships and interpersonal conflicts. In this way, fostering emotional education in the classroom will make students aware of the emotions they feel, and why they feel them, acquire coping skills, make responsible decisions, and fundamentally learn alternative skills to aggression and also prevent and/or manage conflicts. Another fundamental element that should be highlighted is the importance of implementing methodologies that involve emotional education, so that students acquire emotional competencies that lead to emotional regulation, for example of anger, since a part of violence originates from anger; or in other words, the poor regulation of anger is the origin of many violent behaviours.

Despite the results achieved in this study, it is necessary to point out some limitations. (A) The study is based mainly on self-reported scales, with participants being able to make positive attributions to their own results, and negative attributions to events of external forces. (B) The selection of the sample was incidental non-probabilistic, which makes it difficult to replicate the sample. (C) This was a relational study, which did not allow the causality of relationships to be identified. Therefore, the interpretation of results may vary according to each person’s perception. Future studies should analyse the influence of the social and school context on young people’s emotional intelligence and social skills, as well as their influence on peer bullying. In this sense, the family and the social context (e.g., friends, peers, teachers, mass media, etc.) represent a fundamental pillar in the process of the socialization of young people, having a very important influence on the behaviour of adolescents.

## 5. Conclusions

The results obtained in the present study show a positive association of emotional intelligence and social skills, and how these were negatively related to bullying among peers. Finally, emotional intelligence and social skills were negatively related to peer harassment. 

## Figures and Tables

**Table 1:** Adjustment Indexes.

χ2/df (Chi-square Coefficient Divided by Degrees of Freedom)	Values Below 5
TLI (Tucker Lewis Index), CFI (Comparative Fit Index), IFI (Incremental Fit Index)	0.95 or higher
RMSEA (Root Mean Square Error of Approximation) with confidence interval (CI) at 90%	Equal to or less than 0.06
SRMR (Standardized Root Mean Square Residual)	Equal to or less than 0.08

**Table 2:** Descriptive statistics and Pearson’s correlations.

Factors	*M*	*SD*	Range	1	2	3
1. Emotional Intelligence	3.47	1.86	1–5		0.55 ***	−0.31 ***
2. Social Skills	1.59	0.21	1–2			−0.47 **
3. Stalking Behaviour	1.98	0.68	1–3			

Note: *** *p* < 0.001; ** *p*< 0.01.
